# Sensors and Systems for Physical Rehabilitation and Health Monitoring—A Review

**DOI:** 10.3390/s20154063

**Published:** 2020-07-22

**Authors:** Lucas Medeiros Souza do Nascimento, Lucas Vacilotto Bonfati, Melissa La Banca Freitas, José Jair Alves Mendes Junior, Hugo Valadares Siqueira, Sergio Luiz Stevan

**Affiliations:** 1 Graduate Program in Electrical Engineering (PPGEE), Federal University of Technology of Parana (UTFPR), Ponta Grossa (PR) 84016-210, Brazil; lucasnascimento@alunos.utfpr.edu.br (L.M.S.d.N.); bonfati@alunos.utfpr.edu.br (L.V.B.); melissa.1995@alunos.utfpr.edu.br (M.L.B.F.); hugosiqueira@utfpr.edu.br (H.V.S.); 2 Graduate Program in Electrical Engineering and Industrial Informatics (CPGEI), Federal University of Technology of Parana (UTFPR), Curitiba (PR) 80230-901, Brazil; mendes.junior13@yahoo.com.br

**Keywords:** sensors, systems, physical rehabilitation, e-health systems

## Abstract

The use of wearable equipment and sensing devices to monitor physical activities, whether for well-being, sports monitoring, or medical rehabilitation, has expanded rapidly due to the evolution of sensing techniques, cheaper integrated circuits, and the development of connectivity technologies. In this scenario, this paper presents a state-of-the-art review of sensors and systems for rehabilitation and health monitoring. Although we know the increasing importance of data processing techniques, our focus was on analyzing the implementation of sensors and biomedical applications. Although many themes overlap, we organized this review based on three groups: Sensors in Healthcare, Home Medical Assistance, and Continuous Health Monitoring; Systems and Sensors in Physical Rehabilitation; and Assistive Systems.

## 1. Introduction

The recent evolution of electronics and communication systems has been of great importance to the development of comfort devices. Some reasons for this are the easily accessible technology, power systems that allow greater autonomy to devices, and miniaturization of electronic circuits, which are factors that enable the continuous monitoring of individuals [1].

Monitoring and analysis of bioelectrical signals and movements can aid diagnosis, prevention, and examination of a wide range of issues. Remote monitoring and ambulatory monitoring are growing needs in the healthcare environment [2]. These processes pass through stages of pre-analysis and feature extraction considering the applications of, for example, orthopedic, neurological, cardiovascular, and pulmonary problems. From these, it is possible to extract some characteristics that allow for standard setting that aids in diagnostics. Furthermore, the remote monitoring provides increased data volume and for this reason, can promote a better diagnosis for diseases [3], improve athlete performance [4,5], and/or accelerate patient rehabilitation processes [6,7].

In this scenario, wearable devices have great importance in identifying health conditions. They allow monitoring parameters such as temperature, positioning, and electrical bio-signals as electrocardiograms (ECGs), electromyograms (EMGs), and electroencephalograms (EEGs). These are all relevant to medical analysis, personal fit of treatment plans, and monitoring of the patient acquired by the mobile health (m-health) applications [2,8,9].

The advancement of such devices, associated with the increase in Internet of Things (IoT) applications, converges to the concept of electronic healthcare (e-Health). E-Health is the use of electronic devices usually placed or carried next to the patient’s body for collecting and transmitting data to be accessed remotely [10]. With the popularization of Industry 4.0, similarly, the Health 4.0 concept seeks to facilitate the progressive virtualization of individuals, medical devices, and processes for customized online health based on cloud computing [8,11,12]. This concept can provide a solid path to the application of Industry 4.0 concepts such as the Internet of Everything (IoE), wireless communication, and cyber-physical systems (CPS) for solving healthcare problems [13]. CPS contemplates the integration of a virtual universe with physical processes, which are feasible through embedded system monitoring and control of physical processes. Moreover, CPS enables the connection of these two worlds: virtual and physical, in building the IoT that is the basis of Health 4.0 [14].

At the same time, different computational intelligence tools play a fundamental role in providing sophisticated and insightful analyses of the acquired bioelectric and biometric signals including the pre-processing of the signal, extracting characteristics and classifying or clustering them, in addition to statistical analysis.

However, this review aims to analyze, quantify, and emphasize the most widely used sensors and the trend in use for the respective applications. From this perspective, our focus was on sensors and systems (hardware) commonly used in patient monitoring devices and applied to physical rehabilitation, in addition to assistive systems, which reported experimental results.

Thus, this work is organized as follows: initially, an introduction of the theme is presented, underscoring the focus on sensors and justifying this importance. Subsequently, the methodology used in this review is detailed, describing how it was delimited in the research. In Section 3, the review itself is available, subdivided into three topics: Sensors in Healthcare, Home Medical Assistance, and Continuous Health Monitoring; Systems and Sensors in Physical Rehabilitation; and Assistive Systems. Finally, a final review discussion is presented.

## 2. Methodology for This Review

First, we searched for manuscripts across different knowledge fields that had been reported in the last six years. The databases chosen for information selection were related to the engineering and medical fields: PubMed, IEEE (IEEExplore), and Science Direct. We defined five keywords from the main scope and used them to organize this paper: Rehabilitation, Continuous Monitoring, Real-Time Tracking of People with Disabilities, Assisted Living, and Home Health Care. These topics were selected due to being observed in the current applications in biomedical research [15,16].

**Figure 1:**
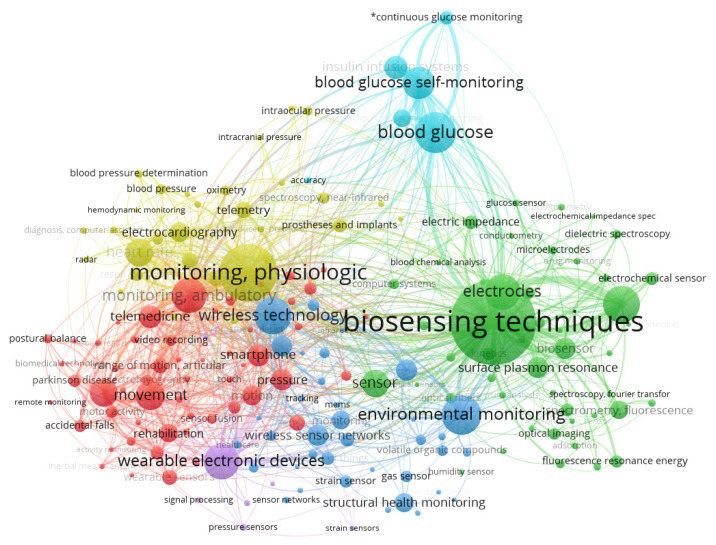
Most recurrent terms provided by VOSviewer bibliometrics software within the following keywords: Sensors and System to Rehabilitation and Caring People with Disabilities.

After the selection of the papers, VOSviewer bibliometrics software [17,18], was used to illustrate the most recurring terms found. As a result of this prior analysis (removing the less relevant terms), Figure 1 presents the occurrence of the main pertinent terms of clusters, indicating a higher presence as a function of the size of every sphere. Six different groups were found: physiological monitoring and telemetry (yellow); correlated glycemic measurements (cyan); bio-sensitivity techniques, electrodes, sensors (green); environmental monitoring and several sensors (blue); telemedicine, movement, smartphone, rehabilitation, and monitoring ambulatory (red); and wearable electronic devices (purple). It is essential to verify in Figure 1 the occurrence of the main terms, but as they are not isolated, arcs indicate that there are correlations between other terms and other clusters.

**Figure 2:**
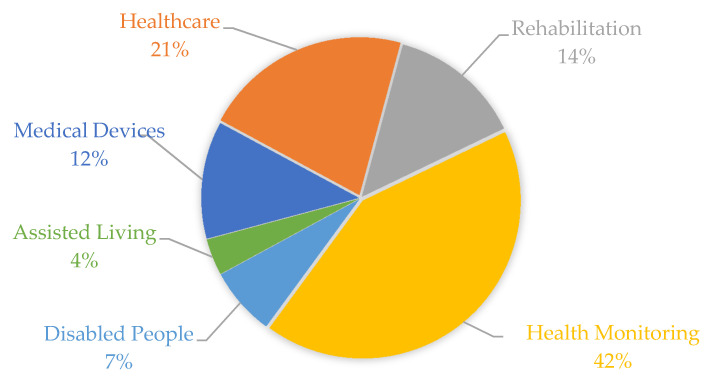
Recurrence proportions of the six terms linked to the keyword “sensor” used to delimit the research results in the IEEE, Science Direct, and PubMed databases from January 2014 to March 2020. This bibliometric analysis was obtained based on more than 13,500 occurrences.

At the same time, several pieces of research were conducted on these scientific bases using as keywords the aforementioned six terms linked to the keyword “sensor” in the delimitated time (the last five years, from January 2014 to March 2020). Figure 2 shows the recurrence proportions of these themes in the collected universe. Healthcare and health monitoring represented about 63% of more than 13,500 occurrences. One of the main reasons for the growth of individual health monitoring solutions, into the hospital or not, comes from the cheaper designs of integrated electronic circuits and the integration of the evolution of IoT and connectivity systems.

**Figure 3:**
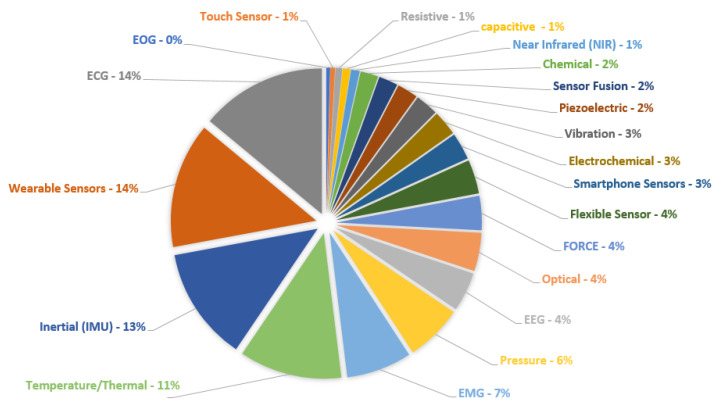
Incidence rate (%) about sensors and techniques found in the IEEE, Science Direct, and PubMed databases using the terms related to the area of Sensors and Systems to Rehabilitation and Caring People with Disabilities, since January 2014 to March 2020.

Furthermore, about 20 keywords were used to correlate the types of sensors and/or their physical quantities to analyze the recurrence of these terms in the researched databases. Figure 3 presents a summary of these terms and their main recurrences over the last five years, in the manuscripts published with the aforementioned themes. A brief analysis showed that ECG, wearable sensors, temperature sensors, and inertial sensors appeared in more than 60% of the selected papers. This significant number can be explained by the increased availability and the lower cost of devices monitoring the patients remotely or monitoring the exercises and daily activities in their routines.

In addition, with the aging of society and the increase in the prevalence of chronic diseases such as cardiovascular disease (CVD), diabetes, respiratory disorders, and others, the demand for continuous monitoring will increase the growth of the wearable devices segment, which will provide greater comfort to the patient and more significant data collection to be analyzed. This, combined with the growing number of mobile devices, has considerably increased the number of m-health devices, whether for medical or fitness purposes. To gain a sense of the financial movement of this market (mobile health), for example, in the USA between the years 2017, 2018, and 2019, it was USD 6.95, 10.2, and 25.17 billion dollars, respectively. In other words, it has almost quadrupled in two years [19].

In this context, about 125 works were selected, divided into the respective subareas of interest mentioned. The criteria for this selection, in the first moment, were based on the combination of the keywords described above, used to filter papers in the main scientific databases. The research period was concentrated on the last six years. Subsequently, the relevance of each work was assessed by analyzing the quantitative and qualitative results presented. It should be noted that works without experimental results and/or purely descriptive results were neglected.

## 3. Sensors and Systems for Rehabilitation and Health Monitoring

This review is subdivided into three major topics to better present and correlate the studies:

(a) Sensors in Healthcare, Home Medical Assistance, and Continuous Health Monitoring, wherein we present the most recent and relevant sensors and systems used for personal health care, home health monitoring, and continuous and/or real-time monitoring applications. Moreover, we consider wearable devices, systems for monitoring patients at home, or athletes during the execution of exercises as part of this theme;

(b) Physical rehabilitation sensors and systems wherein we focus on health care applied in rehabilitation and;

(c) Assistive systems where the main studies that assist in the communication and/or displacement of people with mobility or communication problems (e.g., expression, gesture, and communication recognition devices) are presented.

It is essential to note that from some broader perspectives, there may be an overlap between these three sub-areas, but that we were careful to maintain an organization framework. For example, devices and techniques to aid in rehabilitation activities and exercises were grouped in topic (b), avoiding here the mention of continuous home monitoring techniques. Some monitoring situations such as falls were organized and reported in group (a) for continuous clinical or home monitoring and medical assistance. Likewise, the sensors that appeared in descriptions of assistive systems did not appear in sensors used in healthcare, thus avoiding duplication.

### 3.1. Sensors in Healthcare, Home Medical Assistance, and Continuous Health Monitoring

**Figure 4:**
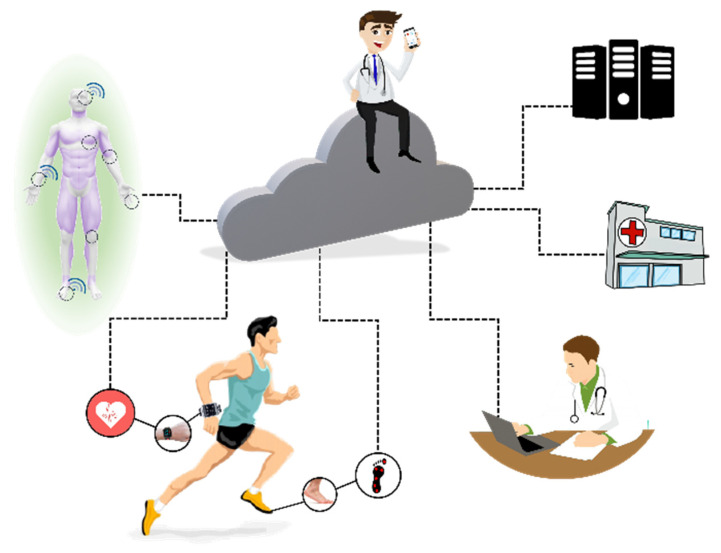
Health 4.0 concepts: IoT and new measurement technologies make the customizing of health possible. Monitoring of patients or athletes is integrated by cloud computing, data collection, and sending it to servers connected to doctors, hospitals, or health professionals. Each sensor can behave as a thing (using IoT concept), and a body area network (BAN) is responsible for communicating with the network (the sensors in contact with the subject or data to the server). This process can occur online.

E-Health systems can provide multi-sensor analysis and data fusion, making possible assertive inferences from the evaluation of biomedical parameters. It directly impacts the health field, mainly in domestic situations that demand simultaneous sensors and support for wireless communication [10,14]. Paralleling, continuous medical assistance, and wearable devices have become crucial for monitoring patients undergoing treatment (or monitoring diseases) and athletes during training sessions, based on portable and mobile devices (known as m-health devices, illustrated in Figure 4). In this scenario, and supported by the possibilities of communication, they allow their doctors to carry out analyses and monitoring at a distance.

The health field is continuously evolving along with the development and improvement of new techniques and equipment for assistance and diagnosis. This evolution is intended to assist physicians and other health professionals in the diagnostic procedure, follow-up, and patient care [20], and to look for new ways to lower the cost of the device [21,22,23]. Advances in microelectronics, telecommunications, sensors, and data processing have enabled a significant and far-reaching development of new healthcare technologies and user support devices [24,25]. Wearable sensors are an example of these advancements that present different monitoring functions (e.g., heart rate, blood oxygen saturation, and sleep patterns) [26,27]. Furthermore, smartphone applications are capable of monitoring the user’s performance in physical activities such as walking, running, and cycling [28] including the execution of exercises during the treatment of diseases [29]. More people are gaining access to these devices and applications due to the ease of purchase and reduction in costs over time.

Devices based on assisted living concepts combine modern technologies to improve the quality of life. Such devices help to monitor people in daily activities such as working, walking, and driving, among others [30]. To improve the life quality of the elderly with different disabilities, the development of assisted living environments with home medical assistance has been more frequent. Home health-care systems [31,32], environmental monitoring [33,34], risk prevention [35], and household tasks assistance [36] are some crescent terms.

To ensure patient safety in an assisted living environment, it is important to monitor both the patient and the environment in which they are inserted. In [33], they presented a system that aims to monitor the people’s activity inside a patient’s room. An omnidirectional camera and algorithm were used to track people moving through image processing and evaluated 260 different positions, which presented an average position error equal to 0.39 m. In another study, [34] presented a proposal to improve the safety of older adults with dementia. Using data from passive infrared sensors, humidity, temperature, sound, light, and gas concentration, an intelligent notification algorithm was created, containing an emergency alarm system, which is determined by the sensor data.

Prevention of risk situations is a determining factor for patients with brain disorders. In [35], they developed a system to prevent these types of situations for people with traumatic brain injury when they are cooking. This was based on the acquisition of data obtained from temperature, electromagnetic, motion, load cell, time meter, and flow meter sensors to identify potentially hazardous situations in the kitchen and display warning messages. After this study, it was concluded that to ensure a better functioning of the system, it would be important to implement a priority management mechanism to check potential risk situations. Helping visually impaired people in household chores also fits as an assisted living resource. An assistance system was presented in [36] to solve this problem, which searches to complement features that help the visually impaired people in their homes, thus increasing their independence. Through temperature, movement sensors, microphones, and among other devices, this system performs several tasks such as identifying people approaching, moving people in the environment, sounding for guidance, user interface, and child supervision.

**Figure 5:**
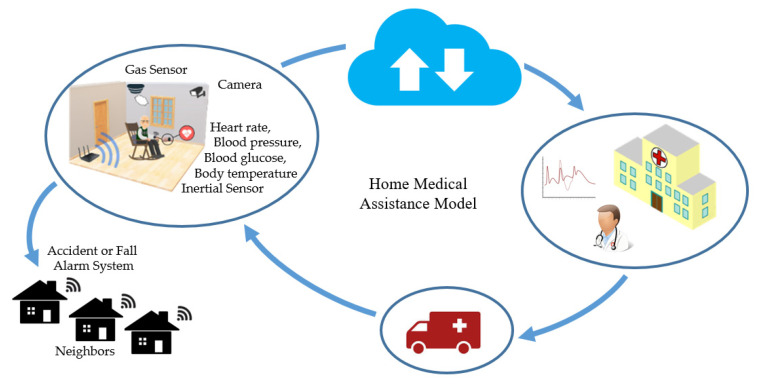
Wearable sensors on patients and environmental monitoring can identify emergencies that trigger only medical care and are directly linked to hospitals and clinics, or connected to a local help network for faster first aid.

In general, elderly and patients with chronic disabilities need constant care to evaluate their health conditions and identify potentially dangerous situations [37,38,39]. This possibility is illustrated in Figure 5. A monitoring system connected to a hospital makes medical assistance at home possible through the IoT concept. Thus, different types of sensors can be used (e.g., GPS receiver, accelerometer, ECG, blood pressure, blood glucose, body temperature, and breathing sensor). In addition to the sensor data, it is possible to resort to online information provided by neighbors and caregivers.

As examples of simplified systems, in [31], the fusion of ECG signals and PPG was reported to provide monitoring and prediction of blood pressure using decision trees models. The pressure accuracy reached above 70% for systolic and 64% for diastolic. The authors claim that the insertion of additional data (e.g., age, body fat ratio, and height) can improve the algorithm accuracy. In [38], aside from using data from sensors such as blood pressure, ECG signal, and body temperature, data acquired from these sensors were sent to an online health monitor through the IoT platform to perform remote patient monitoring. The efficiency of this sensing system was testified, enabling faster access to health and emergency services.

In the study exposed in [40], data collected from the ECG, GPS receiver, accelerometers, and other sensors through a wireless interface allowed for the recognition of possible dangerous situations for patients inside their house and in its surroundings. In this case, 89.1% of precision was obtained for patient detection indoors based on accelerometers and 96.8% for outdoor walking. Additionally, 70.8% of accuracy was obtained when identifying the position of the body inside the house.

Concerning cardiac diseases, in [41], they reported the development of a flexible and wearable biomedical sensor device able to capture bioelectric signals as ECG and EEG based on system-on-chip (SoC) technology. Additionally, the device could measure the temperature due to the SoC concept and had an open-software platform with enhanced connectivity and interchangeability for the integration of devices and services.

In cardiac disorders, monitoring systems can alert the user or even trigger the emergency service autonomously, and can also assist at the hospital, enabling the necessary measures to be initiated in advance. In [42], this study reported a system consisting of a wearable device, smartphone application, and medical station to monitor cardiac patients in rehabilitation. The proposed system enables a patient to perform home-based cardiac rehabilitation exercise based on the interaction between a patient and a specialist anytime and anywhere without visiting a hospital.

The use of ultra-responsive pressure sensors for continuous blood monitoring is presented in [43]. The sensor measures a pulse wave in areas such as the fingertips, wrist, ear, and ankles. Through flexible weaving and plasma etching to generate surface polymer nanowires, this sensor allows for the measurement of a small variation of blood pressure with a sensitivity of 45.7 mV·Pa^−1^.

A system for elderly home care was presented in [37]. This system employed patient-wearable sensors such as GPS, accelerometer, and temperature sensors connected to a mobile device set with information provided by neighbors and caregivers to monitor and create safe spaces. Data were sent to a cloud server to determine the status of the aging patient. Thus, it is possible, through the data provided, to detect emergencies such as if the patient had a fall. In this specific study, it was concluded that the used accelerometer drained the battery quickly, hindering its long-term use for continuous elderly fall detection. Alternatively, the authors proposed the use of the information from GPS to provide tools to check with the neighbors whether something was wrong when the elderly had remained stationary for a long time [37]. In [44], they described an e-Health system for remotely monitoring the health of the elderly through wearable sensors (to measure blood oxygen saturation, skin temperature, and heart-rate, motion sensors using accelerometers, but not limited to these) and operated over the IoT network. The authors analyzed the prototype in terms of latency and lost packages.

In [45], the monitoring of the posture of older persons using wearable sensors based on three inertial sensors was demonstrated. At the same time as they carried out daily activities, they generated indicators to assess their health and quality of life. For this study, thirty independent and semi-independent older persons undertook eight different types of physical activity including walking, raising arms, lowering arms, leaning forward, sitting, sitting upright, transitioning from standing to sitting, and transitioning from sitting to standing. The data were classified offline, achieving an accuracy of 93.5%, and the general perception of the device by the user was positive.

Systems for fall prediction and detection have been reported in [46,47]. To exemplify this, [46] reported a system that monitored four types of falls and eight types of daily living activities with an integrated sensor system. The device used an inertial measurement unit (IMU) and a plantar-pressure measurement unit. The fall-detection performance was evaluated by analyzing the acquired data with threshold detection and the decision-tree method. The results showed that the fall detection algorithm applied to the acceleration and pressure center data in the decision tree method had a 95% accuracy. In [47], a single tri-axial accelerometer attached to the patient’s thigh was used to distinguish between activities of daily living and fall events. The proposed system consisted of a fast mode for fall prediction (event time: from 300 ms to 700 ms) and slow mode for fall detection with a 1 s latency. Using a nonlinear support vector machine (SVM) classifier to identify a fall risk or an event and later alarm the patient, the system obtained accuracy was about 99% for a total number of 600 fall events and activities of daily living (ADLs) cases from 77 subjects.

An alternative way of monitoring and detecting emergencies in patients with chronic disabilities and elderly adults is analyzing their daily routine. The case presented in [48] analyzed the behavior of the patients’ daily routine through passive infrared sensors installed throughout the house. After a long period of monitoring, when the patient behaves differently from this routine, the algorithm may consider this situation as an emergency.

Parkinson’s disease is an illness in which the main motor characteristics are tremors, bradykinesia, rigidity, and impairment of postural balance. Additionally, the patient tends to develop even more motor complications over time [49]. Tracking the development of symptoms can help in prescribing the proper medication. As presented in [50], this monitoring can be performed through the acquisition of data from accelerometers and a system for storing and transmitting the data through the web was used. In this study, a platform was developed to analyze accelerometer data and reliably estimate clinical scores that indicate tremble severity, bradykinesia and dyskinesia. This platform presented three different levels: the first was a resource-aware data collection engine that relies on portable sensors such as the accelerometer; the second consisted of web services for live streaming and sensor data storage, and the third level had a web-based graphical user interface with video conferencing. Data latency results indicated that the proposed platform was suitable for monitoring Parkinson’s disease patients helping drug dose titration in the late stages of the disease.

The monitoring of respiratory muscles is of great importance in critically ill patients. In clinical settings, the main techniques include the use of ventilation flow/pressure sensors; transdiaphragmatic pressure; electromyography; phrenic nerve stimulation, and many imaging techniques (as chest x-ray; fluoroscopy; ultrasonography; computed tomography/magnetic resonance imaging and circulatory biomarkers) [51]. In parallel, some studies have presented some systems for pulmonary monitoring at home, mainly in cases of rehabilitation. The fusion of a wearable device and a motion-sensing exercise monitoring system for pulmonary rehabilitation was intended to measure biomedical data such as heart rate and breathing rate while the patient followed coaching videos for performing physical activity. Data collected from the exercise movements and heart rate of the patient were automatically stored and synchronized with the health management server. Remotely, a medical professional could consult and analyze (online or later) the evolution of the patient’s health status [52]. In [53], a wearable self-powered active sensor for respiration and healthcare monitoring was fabricated based on a flexible piezoelectric nanogenerator, where the respiration signals were measured and compared by a physiological signal recording system.

Alternatively, a study reported breathing monitored remotely through inertial sensors. In [54], a modular system based on IMU (inertial measurement unit) was presented that was composed of three units. Two units were placed on the chest and abdomen to record the respiratory movement and the third unit, which functioned as a central base, was placed on the trunk or in a region integrated with the trunk (for example, support for the back of a wheelchair or bed), but not involved in a breathing movement. The inspiratory time measurements using the proposed device were compared with data from optoelectronic plethysmography and the regression analysis showed that the relative error estimate was less than 5%.

Continuous respiratory monitoring devices (which are an important clinical tool for patients) ensure adequate oxygen supply and indicate cardiovascular diseases, metabolic issues, and sleep problems [55,56]. One of the non-invasive techniques in this monitoring is capnometry, which finds the dioxide carbon (CO_2_) concentration in gas exhaled by the lungs and analyzes the amount of infrared light that is absorbed by CO_2_, where a beam of infrared is necessary. A receiver sensor correlates the gas absorption with voltage in the electronic circuit. Although it is a well-known technique, improvements in this system can be made to measure the CO_2_ concentration accurately, especially for patients undergoing endoscopy [57].

An alternative method to respiratory monitoring fused a piezoresistive pressure sensor and a pyroelectric sensor simultaneously [58]. These sensors measured, respectively, the respiratory flow and the CO_2_ concentration, in real-time. In another study, data from accelerometers distributed on the thorax and airflow signals from a differential pressure transducer in a mask were used for respiratory monitoring [59]. Although it is a classic technique, the use of accelerometers has shown equivalent information for this type of monitoring, especially when the use of such obstructive devices (masks or nasal cannulas) may not be tolerated by critically ill patients. [55]. To monitor patients with chronic respiratory diseases, a wearable system was developed with sensor fusion and low energy consumption [56]. Several parameters were monitored such as ozone level, humidity, photoplethysmography, skin impedance, heart rate, body movements, and a spirometer sensor for analysis of the air expelled by the patient [56].

In [60], they reported a preliminary dynamic sensing device with the potential to change the way clinicians understand disordered gas exchange in patients with lung injury, based on rapid PaO_2_ sensing by a plastic optical fiber sensor capable of being inserted into a standard arterial cannula. The authors also reported the potential to detect an excessive pressure or distension of already aerated lung impairments to blood flow to the ‘ideal alveolus’, allowing better-informed choices of PEEP (positive end-expiratory pressure), respiratory rate, and inspiratory/expiratory (I:E) ratio.

Diabetes monitoring needs to measure the blood sugar level [61]. Usually, an invasive practice is used, which is performed by piercing a finger to extract capillary blood and place it on a reagent strip in a portable device [62]. However, the measures are of punctual and transient behavior, and the scenarios of hyperglycemia and hypoglycemia are lost. As an alternative to this scenario, continuous measurement sensors monitor the concentration of glucose in the interstitial liquid, lasting up to 14 days calibrated [63]. The proposed system can collect commercial sensor glucose rate measurements and display them on a smartphone without scanning manually.

For glucose monitoring, chemical sensors are used (e.g., a sensor based on two enzymatic reactions catalyzed by oxidase and catalase [64]). This smart sensor is implanted in abdominal tissue sites into subcutaneous adipose tissue. It has a microcontroller and a telemetry circuit. The device remained implanted in the subject for 180 days, transmitting data every two minutes, and requiring a monthly calibration to reduce errors, which could vary 2.6% per week. Individual sensor clamps have a correlation coefficient of 0.84 and 0.98 for spontaneous glucose excursions [64].

An alternative smart sensor combines a glucose sensor, temperature measurement, and digital baseband [65]. Three semipermeable glucose layers measure the glucose concentration in subjects using a glucose oxidase enzyme. A potentiostat and a digital-to-analog converter (DAC) are used to reduce device size and battery consumption. The temperature sensor corrects the information of the temperature coefficient in the glucose sensor to provide real-time data. Data transmission is made by the RFID protocol (radio frequency), allowing communication without a battery. Clinical in vivo tests were performed in male Wistar rats, and the device was evaluated with a commercial glucometer with high correlations [65].

A non-invasive method for glucose measurement is the use of photoplethysmography [66]. A device with a near-infrared (NIR) LED (940 nm) near a photodiode is used to measure this signal and to correlate it to the glucose concentration in blood. A model based on exponential Gaussian process regression was used to estimate the glucose level with 97.5% accuracy for 200 subjects [66]. This kind of operation is highlighted as it is a non-invasive technique, unlike the aforementioned applications, which need an implant on the subject.

In another study, monitoring of continuous and longitudinal cerebral blood flow in vivo allows for observing different types of mechanisms and interventions in brain diseases [32]. An infrared laser sensor with a 2D camera can be used to monitor cerebral blood pressure with a speckle diffuse contrast flow meter. This technique presented a high correlation coefficient for both mice (R^2^ = 0.95) and humans (R^2^ = 0.94).

A prototype for a continuous, non-invasive, compact, and affordable blood glucose measurement system based on electromagnetic properties of blood glucose was implemented in [67]. The prototype was implemented in vivo with a Bluetooth low energy module communication. The prototype was validated by simultaneous measurements with the traditional invasive finger pricking technique, presenting a high correlation in the oral glucose tolerant test.

In [68], the authors reported a napkin-based wearable capacitive sensor fabricated by a novel, low-cost, and facile strategy. It is composed of two pieces of electrode plates manufactured by a spontaneous assembly of silver nanowires on a polydimethylsiloxane-patterned napkin. It was demonstrated to have an ultrahigh sensitivity of 17.2 kPa^−1^, and capability for the simultaneous detection of multiple signals. The authors demonstrated that the capacitive sensor could be applied to identify a variety of human physiological signals including finger motions, eye blinking, and minute wrist pulse.

In [69], a flexible and fully integrated sensor matrix was presented for the analysis of multi-complex sweating in situ, which simultaneously and selectively measures sweat metabolites (such as glucose oxidase and lactate oxidase) and electrolytes (such as sodium and potassium ions) as well as skin temperature (to calibrate the sensor response). Accuracy of on-body measurements was verified through the comparison of on-body sensor readings from the forehead with off-body measurements from collected sweat samples. The wearable system was used to measure the detailed sweat profile of human subjects engaged in prolonged indoor and outdoor physical activities, and to make a real-time assessment of the physiological state of the subjects. This platform enables a wide range of personalized diagnostic and physiological monitoring applications.

The works in [70,71,72] have also recently reporting studies toward the development of a continuous and non-invasive sensing device for detecting specific analyses in sweat using electrochemical sensing. In their approach, they have investigated a range of innovative sensing platforms including wristbands [70], stick-on flexible sensors [71], and traditional eyeglasses [72]. These wireless eyeglasses are multiplex chemical sensing platforms for simultaneous real-time monitoring of sweat electrolytes and metabolites [72] and was based on a nose-bridge pad lactate biosensor. For calibration, a commercial glucose meter was used. Both amperometric and potentiometric sensors maintained the high performance of the device.

Different technologies have been developed and applied for monitoring sweating, focusing on spots and the hydration control of athletes. This is the case of a chemiresistor (based on cellulose fibers and carbon nanotubes) used to monitor the sweat rate and the sweat loss for on-body detection of biofluids [73]. These sensors can be applied to aid the monitoring of the hydration levels of athletes, aiming to improve their performance. In addition, the fusion of chemical sensors to detect sweating (tyrosine and uric acid), temperature, and respiration rate could be used to monitor athletes during sports activation and to verify if their diets are efficient [74].

Based on the above, it is evident that the recent growth in the development of wearable devices for the remote monitoring of vital levels such as ECG and PPG signs, blood pressure, blood glucose, chest movement for breathing assessment, among others as well as for the continuous analysis of daily activities, fall detection, or fitness purposes. From this perspective, Table 1 presents a summary of the main sensors and their applications, summarizing this section.

It is also important to note that part of the base of the systems discussed above also allows for its application for people with rehabilitation needs, which provide remote development and continuous monitoring of these activities, providing more continuous and better-applied treatments.

### 3.2. Systems and Sensors in Physical Rehabilitation

Different factors interfere with the physical skills of the person; many of them originate from a muscular injury, a surgical procedure, degenerative diseases, heart disease, and even the age factor [67]. Mobility problems can be caused by neurological [75] and orthopedic dysfunctions (an acquired deficiency, innate deficiency, or incorrect posture) [76]. Rehabilitation is essential for the maintenance of movements or reestablishment of patient function. It aims for a full recovery of these impaired functions and, if it is not possible, it improves the movement of the upper and lower limbs responsible for locomotion, especially the movement of the arms [77].

The authors in [78] reported a methodology for assessing the quality of rehabilitation exercises remotely using IMU sensors and sEMG sensors to track people exercising. The authors reported that the methodology was evaluated with 17 physiotherapy patients, obtaining an average accuracy of 96% in detecting issues such as acceleration, rotation, angular velocity, and posture information in the exercises monitored [78]. In [79], sEMG sensors and accelerometers were used in a wearable application associated with a game-based training system and user feedback system, presenting the results of system performance, game experience, and the training effect.

Many studies have discussed the use of force-sensors to obtain pressure foot evaluation [80,81,82]. In [80], a wearable and unobtrusive system capable of estimating ambulatory gait and balance measures such as the extrapolated center of mass and dynamic margin of stability was reported. The system can tackle impairment in motor ability, gait, and balance in populations such as the elderly, stroke, multiple sclerosis, and Parkinson’s. Different pressure sensor configurations on each foot and an ultrasound range estimation were studied and compared with an Ambulatory Gait and Balance System consisting of 3D F&M (forces and moments) sensors and IMUs, with a mean absolute root mean square (RMS) error of 2.2 ± 0.3 cm. The study showed that pressure sensors, minimally under the heel and toe, offered a lightweight and inconspicuous alternative for F&M sensing toward estimating ambulatory gait and dynamic balance.

**Figure 6:**
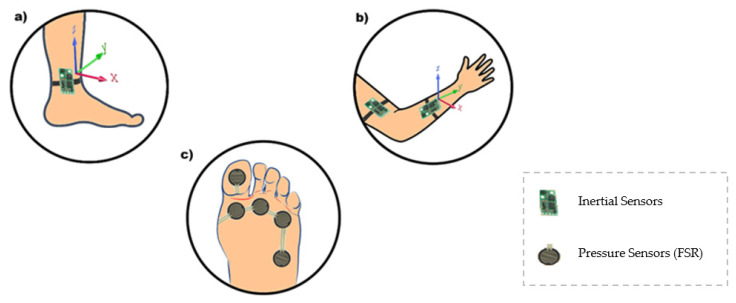
Different sensors can be used in the rehabilitation process. To illustrate this, an inertial sensor can be placed on the patient’s ankle (**a**), while the same inertial sensor can be installed in the arm and forearm (**b**). FSR can also be used to measure foot plantar pressure (**c**).

Inertial sensors and force sensors (FSR) for gait analysis, knee angle, and foot plantar pressure during gait were used in [81]. Foot plantar pressure signals were obtained by an instrumentation insole with six FSRs distributed at some pressure points of the foot, according to movement phases. However, knee angle information comes from four inertial sensors that were positioned one above the knee and one below for both legs. It obtains the limb’s angulation through each sensor and, when combined, can calculate the angle between them. A software collects the data, shows them to users, and analyzes normal and abnormal gait, making it possible to distinguish a significant difference in the minimum angle. Upper and lower limb mobility can be reduced because of an adverse. Figure 6 illustrates a purpose like this, with the use of inertial and/or pressure sensors.

In [83], three different modal time-varying dynamic data, marker trajectory using a six-camera motion capture system, ground reaction force using two force plates embedded in the floor, and eight channels of sEMG signals, were synchronously recorded by a motion capture system from the subjects in both pathological and normal groups. The monitored muscles involved the rectus femoris, long head of the biceps femoris, tibialis anterior, and medial head of gastrocnemius of both lower limbs. The results show that both the recognition and estimation performance reached 98% of accuracy. The authors claim that the results may facilitate the automatic gait analysis system to better support traditional clinical decisions, and thereby enhance the efficiency of rehabilitation treatments.

A stroke (ST) is characterized by an interruption of the blood supply to the brain. There are three main types of stroke: transient ischemic attack, ischemic stroke, and hemorrhagic stroke. It is estimated that 87 percent of strokes are ischemic. It can cause damage that affects different functions such as mobility and speech, and it may be a temporary or permanent condition. For this, systems for lower limb function rehabilitation might be used. Another technique for the rehabilitation of patients who suffered ST based on inertial sensors placed in the ankle is presented in [84]. This device is capable of assisting in diagnosis and rehabilitation in a hospital setting. A multi-sensor fusion algorithm was used to obtain gait parameters, and the transmission was wireless [84]. The verification of the normal and abnormal gait difference was performed by comparing the obtained information and the means of the foot elevation graphs, which makes the asymmetry clearer.

To assess the movement of people that suffered from multiple sclerosis, Parkinson’s, or a ST, a device with inertial sensors was placed on the chest of patients when climbing stairs [77]. Through the statistical analysis of gait characteristics and torso rotation, it was observed that the values indicated that all three pathologies presented low velocity, impaired dynamic balance, and abnormal performance when compared to healthy individuals. A correlation between gait characteristics and torso rotation was statistically verified with clinical evaluations. The use of the sensors showed that these three pathologies presented different behavior regarding torso rotation and rhythm, which was not clinically verified among individuals and may allow a classification between them.

Upper limb rehabilitation in people recovering from a stroke can be performed using inertial sensors as presented by [85], which used two IMUs to increase more movements with appropriate feedback. One of the sensors was installed on the ST affected limb and another on the unaffected one, employing a visual feedback system or vibrotactile that informs the patients that they can use the affected area without receiving negative feedback as often when necessary. After the data were collected and the acceleration preprocessed, seven features were extracted and the algorithms of minimum redundancy (minimum correlation between the features) and maximum relevance (maximum correlation with the movement category) were used to rank the most relevant features. The training of the logistic regression classification model was performed to distinguish goal-directed (GD) movement from non-GD, and the leave-one-subject-out cross-validation technique. The presented results show that it is possible to detect movements during the execution of ADL with an accuracy of 87.0% and detect incorrectly-performed home-based rehabilitation exercises (for example, arm raise) with an accuracy of 84.3% [85].

Weight support systems are commonly used for rehabilitation in patients with an inability to maintain the center of gravity. To reduce the load on lower limbs, facilitate movement, and provide individual sustentation during the gait, a robot was developed to assist in the rehabilitation exercises of lower motor dysfunction [86]. A mechanical voltage sensor was used to provide real-time response to control the robot.

An alternative device to gait rehabilitation is an in-shoe with pneumatic gel muscle [77]. Two measurement techniques were used: an FSR sensor measures the changes in the gain of pressure control according to the step phase, and a sEMG system obtains the amplitude of the activation signals, changing the material rigidity of the gel muscle by air pressure. The system registered a reduction of the load of about 25%.

One of the consequences of stroke is hand impairment, and serious games can be applied to aid patients in rehabilitation [87]. A cursor position was controlled by the sEMG signals acquired from four muscles, providing visual feedback for the patient in five implemented games. This device reduced about 50% of the required time to perform the patient’s activities [87]. Additionally, sEMG devices such as the Myo armband can be used in the rehabilitation process. To assist in the treatment of frozen shoulder syndrome, Myo is used to provide interactive biofeedback in exercises [88].

In [89], the authors reported the manufacturing of textile electrodes to applications based on surface electromyography (sEMG), which is particularly relevant in rehabilitation, training, and muscle function assessment. The electrodes were obtained by depositing poly-3,4-ethylene dioxythiophene doped with poly(styrene sulfonate) onto cotton fabric and then selectively changing the physical properties of the textile substrate. The validation was based on their functional and electrical characteristics. They were assessed for two different electrode sizes and three skin-interface conditions (dry, solid hydrogel, or saline solution) and compared to conventional disposable gelled electrodes. The comparison yielded a correlation value higher than 97% for all measurement conditions, even in situations of intense physical activity.

Virtual reality (VR) is a powerful tool in the rehabilitation process of brain injury by ST [90], accelerating the recovery of the patients. VR reduces the need for hospitalization and stimulates several regions of the brain, inducing the repair of the compromised neurons. This technology uses a Kinect^®^ sensor to capture images and a Leap Motion sensor to track hand movements. Thus, two training systems were applied to reduce the time to finish the activities and to improve finger mobility dysfunction and balance impairment [90]. Another example is the use of VR in a serious game based on an exoskeleton for stroke patients with upper limb motor dysfunction [91].

Many studies highlight VR as an essential auxiliary technique when coupled with motion sensing and monitoring systems [92,93]. In [92], the authors conducted a study based on VR to investigate rehabilitation measures using a football game as a tool for children with cerebral palsy, spinal cord injury, multiple sclerosis, and other neurological disorders and in healthy individuals. The hip and knee were equipped with force sensors, which measured the force necessary to keep the subject in the predefined gait trajectory.

The use of robot-assisted gait training has been one of the most current studies in the field of rehabilitation, showing itself as a promising area [94,95,96,97]. In [98], the authors reported an initial study to analyze the emotion of patients, offering a therapist a better interpretation of the psychological state of the patient and their transition from each emotional state. In this analysis, they used EEG signals, PPG acquired from a wristband, a 3-axis accelerometer to capture motion-based activity, an electrodermal activity sensor to measure sympathetic nervous system arousal and related features such as stress and excitement, an infrared thermometer, and an event mark button to tag events and correlate them with physiological signals.

In [99], the authors reported the development of a portable device for a training technique that typically increases the vestibulo-ocular reflex (VOR) response a minimum of 15% after 15 min of training. The device consists of two parts. The head unit contains inertial sensors to measure the instantaneous 3-D orientation of the head in space at 250 Hz, and an integrated circuit mirror to dynamically control the position of a laser target in space. The base unit consists of a touch screen interface that allows users to calibrate and set the device, in addition to recording compliance. The laser target range was ±12.5°, with a latency of 6 ms with a frequency response stable up to 6 Hz for velocities higher than 80°/s. In experimental tests, 10 normal subjects presented VOR increased by ~11%. In clinical use, patients can train regularly at home as part of a rehabilitation program.

In [100], the authors reported that a multi-sensor fusion between the Kinect camera and inertial sensors, focusing on upper and lower limbs, was developed to estimate kinematic data from different joints for rehabilitation and clinical monitoring. The system showed that multi-sensor fusion provides useful data for clinical follow-up, being a solution for home rehabilitation.

Based on all the content discussed in this section, it is clear that when it comes to rehabilitation, it is about monitoring the human body and its dynamics to support exercises and clinical and physical therapy interventions. For this purpose, bioelectric sensors, especially for electromyography, added to inertial sensors and pressure sensors are widely used to control movements and evaluate muscle. Furthermore, several motion monitoring sensors (infrared, ultrasound, depth sensor, camera, and multi-array microphone, etc.) are commonly found, as summarized in Table 2. In addition to these, stimulus systems such as Virtual Reality and Serious Games constantly appear as auxiliary platforms for rehabilitation.

Invariably, these systems can allow for the local assessment as well as the remote assessment of the patient, based on the same concepts of the m-health devices already discussed; likewise, they can not only allow physical rehabilitation, but also similarly promote assistive devices for people with motor limitations.

### 3.3. Assistive Systems

Regarding sensors and systems for health care and monitoring, this section seeks to correlate the most recent reported studies with the focus on auxiliary devices for people with motor restrictions and also on devices to assist communication, whether through pattern recognition systems in communication (such as gestures and eye movement) or systems of understanding feelings and expressions (facial).

**Figure 7:**
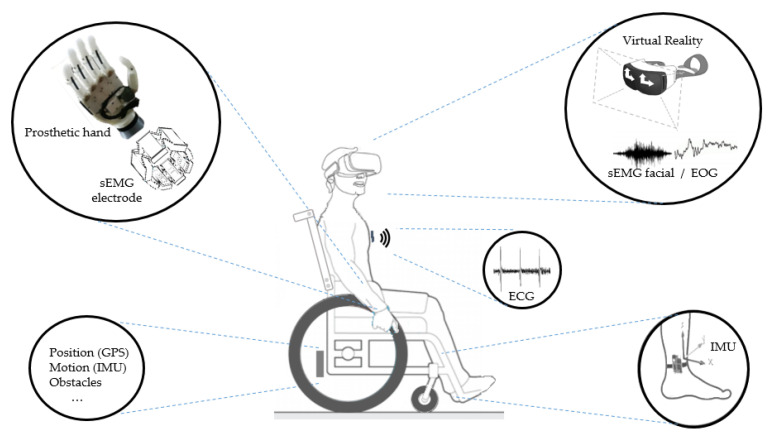
Different sensors can be used in the assistive systems. To illustrate this, a wheelchair is shown with different applications: Prosthetics with sEMG sensor; Glasses with sEMG and EOG sensors; Devices to monitoring vital signs as ECG, and Inertial sensors to monitoring the user’s movements and wheelchair movements.

To illustrate the above, Figure 7 shows some possibilities of assistive systems, initially based on a subject with limited mobility. As a main element of the figure, a wheelchair illustrates the possibilities of sensing locomotion and obstacles and, in addition, we can imagine the possibilities of control by voice command. In parallel, a set of sensors on wearable devices can be coupled to the user in different ways. For example, sensors for monitoring vital signs, whether for movement analysis of limbs or head or for prosthesis control by EMG, or even through devices attached to the head such as virtual reality glasses or other wearable elements for signal acquisition of facial sEMG, EOG, or EEG.

In [101], the development of a new domotic healthcare system for patients with amyotrophic lateral sclerosis (ALS) to improve home-based clinical follow-up through the identification of functional loss worsening and complications was, preserving interaction between patients and caregivers. Using electromedical wireless sensors, the authors enveloped a device to analyze breathing, nutrition, motility, and communication: pulse oximeter, sEMG sensor associated with a dynamometer, laryngophone, and EEG-based brain-computer interface (BCI) device. Visual analogic scale (VAS) and system usability scales (SUS) were used to analyze the user-friendliness and the satisfaction of each sensor. The domotic interface was tested on eight patients with ALS. The authors reported that the SUS and VAS scores indicated a good system usability, both in satisfaction and ease of use.

In [102], the authors reported the design, development, and validation of the eyelid drive system (EDS), an assistive technology comprising a specialized pair of glasses and millimeter-sized passive resonators attached to the wearer’s eyelids that transduced eyelid movement (blinking and winking) through inductive sensing. Initial trials were performed involving six human subjects, and a group meant that the accuracy of 96.3% was achieved using a set of four different commands at a response rate of 3 s. The authors concluded that related eye-interfacing assistive technologies provided advantages such as high accuracy, wearability, insensitivity to lighting and noise conditions, aesthetic acceptability, and use of non-exaggerated gestures.

In another study, [103], a speller BCI system consisting of a virtual keyboard based in EEG signals was reported. It consisted of EEG acquisition and real-time processing equipped with separate keyboard-display control, optimal stopping of flashes, and word prediction. Twenty ALS patients participated in the experiment. The system was effective in that all participants successfully achieved all spelling tasks and was efficient in that 65% of participants selected more than 95% of the correct symbols. The authors reported the mean number of correct symbols selected per minute ranged from 3.6 (without word prediction) to 5.04 (with word prediction).

In [104], the authors reported a comparative analysis from the standard individual arm therapy and set of four assistive therapy systems (three robotic devices and one multi-sensor device). It was composed of a robotic device that allowed for passive, active-assistive, and active finger flexion and extension movements; a robotic device that allowed for 3-dimensional unilateral or bilateral movements of the shoulder joint with arm weight compensation against gravity; a robotic device that allowed for passive, active-assistive, and active planar movements of the shoulder and elbow joints; and a multisensory-based system that allowed for unassisted unilateral or bilateral 3-dimensional movements of the shoulder, elbow, and wrist joint. Sixty patients with upper limb impairment caused by neurologic (91.7%) or orthopedic (8.3%) disorders were studied. The analysis of the data collected with the multisensory system allowed for the conclusion that robotic group therapy could cost less than half a session than the equivalent results obtained by standard individual arm therapy.

Assistive technologies that were previously restricted to medical centers are available in mobile devices [41] including sensor gloves, wristlets, shoes, glasses, and other devices to promote health monitoring and remote medical assistance. To exemplify, the work in [105] presented a soft robotic glove designed to assist individuals with functional grasp pathology to perform activities of daily living. Surface electromyography (sEMG) and radio-frequency identification techniques were adopted to detect user intent to activate or deactivate the glove and pneumatic actuators [105]. Thus, in [106], they reported an inductance-based flexible pressure sensor to support the tactile communication between deafblind people, which is intended to be integrated into a smart glove with the dual function of tactile sensing and vibrotactile feedback [106].

In [107], the authors reported that a sensor glove employed for human-robot interaction in a robotic assistive system was designed, produced, and evaluated. The sensor glove was based on flex sensors, which were utilized to detect the user’s finger gestures and IMU, which was used to detect the arm movements of the user. In this study, eight finger signs were recognized with an average hit rate greater than 98%.

To locate the user’s iris, showing sensitivity to distance changes and glasses, a Kinect^®^ camera was used. The Kinect^®^ camera has RBG-D sensors that provide color and depth of synchronized images, and it was possible to locate the iris with an accuracy of 98.6%, and even a head rotation angle of up to 180° and a 15° head deviation [108].

Head-neck interfaces have the potential to command and control orientation tasks when the hand-wrist is not available for use as a joystick. In [109], the authors reported the use of a human-like avatar to analyze the trajectories of the head based on actual positions and target in a visual interface (computer screen). The robotic neck brace was validated for both head motion measurements and for providing physical assistance through servomotors. The sketch used the installation of the six-axis force/torque sensor, and encoders and current sensors were housed in these servomotors. The outcomes were defined by comparison between the head-neck and hand-wrist motions as follows: (1) mean absolute error; (2) time delay in tracking continuous orientation trajectories; and (3) settling time to reach target orientation. The authors related that the performance outcomes were significantly better with the hand-wrist than that of the head-neck when used as a joystick. However, all participants completed the tasks with the head-neck. This demonstrates that the head-neck can be used as a joystick for controlling three-dimensional object orientation, even though it may not be as skillful as the hand-wrist.

Regarding human-machine interfaces for controlling wheelchairs, there are several methods in the literature. An example presented in [110] was the use of a camera acquire images and the processing tracking face inclination and mouth shape to turn the wheelchair left or right and set a chair in motion or stop, respectively. The device was tested in 34 subjects, and it had a sensitivity for minimal user movements, robustness to ambient brightness, and accurate recognition of user intents [110].

A wheelchair that can control and recognize the environment and obstacles can be constructed by fusing data from a camera and ultrasonic sensors [111]. This chair had 98.3% accuracy in recognizing outdoor locations and 92% accuracy in picking pathways to avoid obstacles, reaching a high level of satisfaction from subjects. Additionally, bioelectrical signals can be used to control the wheelchair such as electrooculogram (EOG) and EEG signals [112].

Several studies relate the use of EOG for the simple control and aid of people with motor restrictions. However, the evolution of accessibility systems seeks to promote a greater number of communication symbols such as the work using EOG to work on the alphabet [113].

In parallel, the work in [114] reported a device with 14 electrodes to capture sEMG signals from facial movements with two added channels of a gyroscope (horizontal and vertical), intending to obtain movement coding for the alphabet. The authors reported a performance rate greater than 83% in capturing signals during the “typing” process.

Seeking to identify facial expressions, the study in [115] proposed an initial system that combined facial expression detection using sEMG with balcony detection through EOGs, in addition to using an inertial sensor to classify, in formal form, four types of expressions: neutral, sad, happy, and angry. The authors showed no analysis of performance and/or statistics.

In [116], the authors presented an asynchronous speller for Chinese sinogram input by incorporating EOG into the conventional EEG-based spelling paradigm. An EOG-based brain switch was used to activate a classic row-column P300-based speller only when spelling was needed, enabling asynchronous operation of the system. This traditional system was also used to verify the performance. The proposed system showed a mean communication speed of approximately 2.39 sinograms per minute, an increase of 0.83 sinograms per minute compared with the P300-based method. The preliminary online performance indicated that the proposed paradigm is a very promising approach for use as an assistive communication tool.

Emotion recognition is also very important, especially when the subject has some disability like autism spectrum disorder (ASD) and presents a difficulty for social interaction. To assist people who are not able to communicate and need a caregiver, a system was developed to detect four different emotions based on information provided by a camera [117]. The system recognized when the subject was angry, sad, happy, or scared with 89% accuracy. Additionally, the system presented high accuracy for the “angry” and “scary” classes due to them presenting significant facial expressions. Another example involved with ASD employed emotion recognition, face-to-face, eye contact, and behavioral self-regulation by using a smartphone with integrated sensors. The sensors captured movement, physiology, application performance, video, and audio for games that recognize the face and emotions. Users and their caregivers completed the intervention session without any adverse effects in the tests [118].

The recognition of emotions based on neural signals is a promising technique for detecting patients’ emotions and improving medical care. However, emotion-related neural signals such as functional near-infrared spectroscopy (fNIRS) can be affected by various psychophysiological and environmental factors, resulting in data instability and classification instability, and this phenomenon can lead to dissatisfaction and abandonment of the system. In [119], the authors proposed a method to mitigate the instability of the classification in recognition of emotions based on fNIRS, using the selection of characteristics for stable characteristics. Preliminary tests showed that this method led to an improvement of approximately 5% in accuracy. Although the current application of fNIRS in rehabilitation and medical care is limited, the authors believe that this approach has a promising future.

In [120], the authors presented an assistive robotic arm controlled through a low-cost combination of stereovision (an RGB-depth camera was chosen instead of active depth sensors) and eye-tracking algorithm. The results presented three scenarios. In the first, a user was able to control the robotic arm, successfully reaching and grasping an object in 92% of the trials without obstacles with an average time of 13.8 s. In the second scenario, with one obstacle, it had a success rate of 91% with an average time of 17.3 s. Finally, in the last case with two obstacles, it had a success rate of 98% with an average time of 18.4 s.

The authors in [121] proposed a device to solve the problem of moving and navigating for visually impaired and blind people. The prototype employed many ultrasonic distance sensors (US), a microcontroller to determine the location of the obstacles according to the US sensor ID, and a voice storage circuit (to tell a blind person where the obstacles are).

Aid communication between people is a topic related to assistive technologies and a class of devices had gesture recognition as the focus. Among them, several works had sign language as a class of gestures to be recognized. These systems presented different types of hardware data processing strategies to solve this problem due to each nation having its own sign language set. In [122] and in [123], the authors developed a similar study focused on a glove-based device fusing data from flex sensors, pressure sensors, and IMUs with Bluetooth communication that was able to recognize the alphabet signs from American Sign Language. The authors in [123] reached 98.2% of accuracy using a support vector machine classifier, while in [122], they achieved 96.5% of accuracy by applying a Kalman filter and using dynamic time warping and nearest mapping algorithm in a Raspberry Pi platform. An alternative glove was presented by the study in [124] for sign language recognition using accelerometer sensors. Combined with hidden Markov models with the proposed system, the authors obtained accuracies of 99%.

Furthermore, sEMG devices have been applied for sign language recognition with wearable devices. The work in [125] used a commercial sEMG armband, Myo, to recognize static signs for the Brazilian Sign Language alphabet. The authors used a neural network classifier and obtained accuracies above 80%. The hit rates increased by fusing sEMG data with IMU sensors, as demonstrated by [126]. The author fused sEMG and accelerometer sensors, who observed that combining these data increased the accuracy in the classification of 10 signs for Indian Sign Language. In another case, the study in [127] combined signals for sEMG and IMU sensors to recognize 80 signs for American Sign Language based on SVM with accuracies above 90%.

New technologies for the development of sEMG sensors have been applied in assistive technologies. For example, a capacitive sensor was developed by the authors in [128], which described an electrode that could be used in the skin of subjects with circulatory disorders that did not need skin preparation. This kind of sensor can be applied for prosthesis control. Another example is the development of a polymer-based electrode for sEMG detection with low cost, low noise level, and high correlation to the signal (in order of 97%) [89].

In this context, we found that assistive systems were formed by different sensors with the essential objective of seeking to increase autonomy for people with some motor limitations. Thus, the monitoring of bioelectric signals (EEG, ECG, EMG, and EOG) often added to inertial sensors is essential, especially for newly operated or paraplegic patients, as summarized in Table 3. Furthermore, we verified a wide range of studies on the movement, gesture, and alphabet recognition system, which could also promote a better interface (man-computer) with various equipment.

## 4. Discussion: Limitations and Perspectives

The main contributions of this article are: first, the presentation of a dense set of highly dispersed works in the biomedical area, mainly focusing on information about sensors and systems for physical rehabilitation and health monitoring; second, promote the indication of the main types of sensors currently being used for biomedical applications; and, third, to point out that there is an increasing evolution in the development of wearable devices, especially with a view to the concepts of e-Health and Health 4.0, stimulated by the recent development of embedded processing and communication devices as well as technologies such as IoT and cloud and fog computing for health in vision.

In this scenario, we have noticed the growing number of medical and fitness monitoring devices appearing on the market in recent years. Financial reports point to continued growth in investment in this sector, especially in the wearable medical devices market.

The wearable medical devices being developed not only track activity (e.g., for fall detection or activity characterization) including heart rate, steps, and calories burned, but also have sensors that pick up vital signs such as respiration rate, blood-oxygen levels, irregular heartbeats, pressure plantar, muscular activity, and others.

The availability of the smartphone and wearable sensor technology is leading to a rapid accumulation of human subject data, and machine learning is emerging as a technique to map these data into clinical predictions. At-risk patients such as elderly people with diabetes, asthma, chronic obstructive pulmonary disease, and heart irregularities have a lot to gain from these wearables. The possibility of the continuous collection of different sensors can allow for more reliable medical evaluation at a distance, promoting online prescriptions, and reducing the frequency of face-to-face consultations.

Advances in health technology as well as parallel technologies such as big data, cloud computing, artificial intelligence, among others, are resulting in the continuous improvement of treatments and have a fundamental role in preventing diseases or their sequels, taking into account the medical history, a person’s environment, and lifestyle.

It is notorious that different manufacturers have their devices based on distinct technological qualities and operate under different software. Furthermore, the accuracy and types of data collected by these devices are not the same. There is a lack of regulatory controls, which makes them difficult to use in more comprehensive healthcare and well-being applications. Therefore, more work is needed to determine exactly which parameters should be normalized to affect positive changes in the e-Health industry.

The first consequence of the lack of regulatory controls has to do with quality data security. Since several pieces of equipment do not have the same precision result, or for some reason they may offer inaccurate data, there may be an unintentional suggestion of harmful/inappropriate treatment. Thus, many of these variables can result in inconsistencies in data collection, which may result in an incomplete or imprecise profile of users and, consequently, impair the usefulness of these data by health professionals, especially in a more comprehensive view.

As an example of the security problem, if a device produces false-positive readings, doctors may recommend potentially harmful treatments. A good example could be a reading of blood glucose too high, which read in the wrong way could lead to distance medical advice making an intervention to correct the blood glucose with a fatal amount of insulin.

As an economic consequence, imagine that a set of health status information is collected and that an insurance company can use this information. Poorly collected information may erroneously indicate that an individual may be more likely to have a sudden illness or stroke, which could be used to increase the premium values for this group of people.

On the other hand, although it was not the focus of this work, we know that data processing is a fundamental stage of biomedical monitoring devices, and is continuously evolving, requiring more powerful hardware. This is a significant limitation when it comes to remote medical devices and/or patient monitoring, which implies the need for new software update policies to be implemented by manufacturers.

## 5. Final Considerations

In this review, our focus was to address sensors, devices, and systems commonly used in disease devices and/or patient monitoring of the most recent methods applied for physical and motor rehabilitation that showed experimental results, with the difficult task of neglecting the first focus, works based exclusively on artificial intelligence or advances in networks.

Initially, we performed the analysis of themes and keywords using three important databases in the last six years: PubMed, IEEExplore, and Science Direct. In this selection, the terms “health monitoring”, “medical assistance”, and “rehabilitation” appeared in 42%, 21%, and 14%, respectively. In a second analysis, we found that ECG, wearable sensors, temperature sensors, and inertial sensors appeared in more than 60% of the selected works.

Based on this, to better organize the text, the review was subdivided into three topics: Health Sensors, Home Medical Assistance, and Continuous Health Monitoring; Systems and Sensors in Physical Rehabilitation; and Assistive Systems. The first addressed the possibility of remote monitoring (m-health; e-Health devices) with a focus on prevention and remote assistance. The second sought to present the current sensors and devices for rehabilitation, local or remote. Furthermore, the latter discussed many works of sensors and systems to help people with disabilities or limitations, in addition to, for example, expression, gesture, and communication recognition devices.

The vast majority of sensory devices point to wearable equipment that increasingly allows for continuous data collection. Some studies point to a growth of up to 20% in the use of wearable devices that provide the collection of some biomedical data. Already among researchers, there is a concern not only with new sensing platforms, but also with the increasing insertion of sensor fusion devices. These seek not only to monitor the daily lives of elderly people, but to effectively develop technologies to monitor and assist chronic diseases such as cardiovascular diseases, diabetes, and respiratory disorders, among others, which also reinforces the growth in the segment of portable devices.

This is notorious when we analyze in parallel the development of communication systems and information technology such as the Internet of Things (IoT), cloud and fog computing, and Health 4.0.

However, the greater volume of work related to remote health monitoring topics has shown the constant evolution of sensor data processing techniques, data availability techniques (IoT), and application of artificial intelligence tools, to the detriment of the focus on the development of new sensors, which is important, but has been the subject of fewer reports.

For this reason, the constant evolution of electronic device technology and the evolution of information technology support the growth of connectable devices and the multiple monitoring of health and behavior, resulting in the forecast of abrupt growth in financial investment in these areas, emphasizing the importance of these works.

Furthermore, it was noted in this review that countless studies are increasingly reporting the use of rapid prototyping devices to obtain low-cost systems. However, most investigations found in this regard were concerned with reporting the prototype itself, instead of characterizing and/or critically analyzing the results obtained, and for this reason, were deleted from this review.

At the same time, important data security issues need attention. In particular, we emphasize that the lack of standards and the growing number of manufacturers requires attention so that the collected data are reliable and allow for the performance of supervised clinical actions, but do not lead to divergences in analyses, prescription failures, or inadequate alarms.

## Figures and Tables

**Table 1:** Summary of the main technologies discussed in Sensors in Healthcare, Home Medical Assistance, and Continuous Health Monitoring.

Sensors in Healthcare, Home Medical Assistance, and Continuous Health Monitoring		
Main Application	Sensors	References
Fall detection and posture monitoring for elderly, patients with Parkinson’s disease	Inertial/plantar-pressure measurement unit	[45,46,47,50]
Assisted Living for elderly/patients with chronic disabilities/impaired people	Wearable sensors: ECG/EEG/GPS/inertial/temperature/blood pressure	[37,38,40,42]
	Ambient sensors: Infrared/humidity/gas/light/temperature/camera/movement	[33,34,48]
Respiratory monitoring	Pressure transducer/infrared/piezoresistive pressure/pyroelectric/inertial/PPG	[56,58,59]
Blood monitoring	PPG/infrared/pressure/camera	[13,31,43]
Glucose monitoring	Chemical/glucose/PPG	[61,62,63,64,65,66,67]
Sweat monitoring	Metabolites/electrolytes/skin temperature/electrochemical/stick-on flexible sensor/eyeglasses	[69,70,71,72,73,74]

**Table 2:** Summary of main technologies discussed in Systems and Sensors in Physical Rehabilitation.

Systems and Sensors in Physical Rehabilitation		
Main Application	Sensors	References
Gait analysis and pressure foot evaluation for patients with multiple sclerosis, Parkinson’s disease, patients that suffered from a stroke	Pressure/inertial/FRS force sensor/camera/EMG	[80,81,83,92]
Evaluation of rehabilitation exercises, assess and increase more movements for patients with palsy, Parkinson’s disease, multiple sclerosis, stroke, brain injury	Inertial/EMG/kinect/leap motion sensor	[78,79,89]
Rehabilitation exercises analysis	Inertial/kinect	[99,100]
Use of VR in rehabilitation	Kinect/leap motion sensor/force	[90,91,92,93]

**Table 3:** Summary of main technologies discussed in Assistive Systems.

Assistive Systems		
Main Application	Sensors	References
Movement coding for control keyboards and displays for patients with ALS and people with upper and lower limbs palsy	EEG/EOG/facial EMG/inertial	[103,113,114]
Control and implementation of tasks and human-machine interfaces like wheelchair, smart shoe, and robot	Inertial/flex sensor/camera/ultrasonic/EOG/EEG/Kinect/force/torque/FRS/infrared	[107,108,109,110,111,112,120,128]
Emotion recognition for patients with palsy, autism spectrum disorder	Camera/movement/sound/infrared	[115,117,118,119]
Gesture recognition for aid communication between deaf people and listeners	Flex sensor/inertial/EMG	[122,123,124,125,126,127]
